# *Brachypodium distachyon* T-DNA insertion lines: a model pathosystem to study nonhost resistance to wheat stripe rust

**DOI:** 10.1038/srep25510

**Published:** 2016-05-03

**Authors:** Tianyue An, Yanli Cai, Suzhen Zhao, Jianghong Zhou, Bo Song, Hadi Bux, Xiaoquan Qi

**Affiliations:** 1 Key Laboratory of Plant Molecular Physiology, Institute of Botany, Chinese Academy of Sciences, Beijing, 100093, China; 2 University of Chinese Academy of Sciences, Beijing, 100093, China; 3 Institute of Plant Sciences, University of Sindh, Jamshoro, 76080, Pakistan

## Abstract

Wheat stripe rust, caused by *Puccinia striiformis* f. sp. *tritici* (PST), is one of the most destructive diseases and can cause severe yield losses in many regions of the world. Because of the large size and complexity of wheat genome, it is difficult to study the molecular mechanism of interaction between wheat and PST. *Brachypodium distachyon* has become a model system for temperate grasses’ functional genomics research. The phenotypic evaluation showed that the response of *Brachypodium distachyon* to PST was nonhost resistance (NHR), which allowed us to present this plant-pathogen system as a model to explore the immune response and the molecular mechanism underlying wheat and PST. Here we reported the generation of about 7,000 T-DNA insertion lines based on a highly efficient *Agrobacterium*-mediated transformation system. Hundreds of mutants either more susceptible or more resistant to PST than that of the wild type Bd21 were obtained. The three putative target genes, *Bradi5g17540*, *BdMYB102* and *Bradi5g11590*, of three T-DNA insertion mutants could be involved in NHR of *Brachypodium distachyon* to wheat stripe rust. The systemic pathologic study of this T-DNA mutants would broaden our knowledge of NHR, and assist in breeding wheat cultivars with durable resistance.

Wheat stripe rust, caused by the obligate biotrophic fungal pathogen, *Puccinia striiformis* f. sp. *tritici* (PST), is considered to be the most devastating foliar disease and the most serious threat to global sustainable wheat production^1^. At present, the disease is controlled mainly through the application of fungicides and the genetic deployment of resistant genes that can confer durable resistance to stripe rust in wheat cultivars^2^. However, due to the large size and complexity of wheat genome, it is difficult to clone durable resistant genes and verify the molecular mechanism of interaction between wheat and PST. And only countable studies have been tried to identify genes that maybe be involved in the compatible response. The partial resistant gene *Yr36* is identified by map-based cloning, and is reported to confer resistance to a broad spectrum of stripe rust races at relatively high temperatures. This gene, *WKS1*, includes a kinase and a putative START lipid-binding domain, which is present in wild wheat and could be used to improve resistance to stripe rust in a broad set of varieties^3^. And later the WKS1 was found to target to the chloroplast where it phosphorylates the thylakoid-associated ascorbate peroxidase (tAPX) and reduces its ability to detoxify reactive oxygen species and contributes to cell death^4^. Another partial resistant gene *Yr18*, resembling adenosine triphosphate-binding cassette transporters of the pleiotropic drug resistance subfamily, supports durable resistance to multiple fungal pathogens in wheat including PST^5^. Four resistance quantitative trait loci (QTLs) were detected in the wheat recombinant inbred population derived from a cross between Yanzhan 1 and Xichang 76–9 cultivars, in which the major one, *Yrq1*, was mapped on chromosome 2DS^6^. A wheat *NAC* gene, *TaNAC4*, is found to function as a transcriptional activator involved in wheat response to PST^7^. TaADF7, an actin-depolymerizing factor, modulates the actin cytoskeletal dynamics to influence reactive oxygen species (ROS) accumulation and the HR^8^. A series of host-related genes have also been isolated from the interaction between wheat and PST, such as BAX inhibitor-1^9^, *TaMCA4*^10^, *TaHSC70*^11^, *TaRAR1* and *TaSGT1*^12^. Attempts to clone durable resistant genes have been made in the past several years, but the mechanism underlying durable resistance, especially quantitative resistance, is still largely unknown^13^.

*Brachypodium distachyon*, a temperate monocotyledonous grass, is in the evolution of the Pooideae diverged just prior to the clade of “core pooid” genera that contain the majority of important temperate cereals^14^. Coupled with the biological characteristics it possesses, such as small plant size, short life cycle, highly efficient transformation system and fully sequenced small size genome, it has been proposed as a new model species for functional genomics in temperate grain cereals, including wheat and barley^15^,^16^,^17^,^18^. Due to the close relationship to major cereal crops, *Brachypodium distachyon* could serve as a nonhost and show resistant symptoms to selected cereal pathogens^14^,^19^. Nonhost resistance (NHR) is a resistance that is exhibited by an entire plant species to all genetic variants of a non-adapted pathogen species and represents the most robust and durable form of plant resistance in nature^20^. Increased evidence has shown the viability of *Brachypodium distachyon* as nonhost to commit NHR studies to some selected cereal pathogens, with the unique opportunities its small compact sequenced genome provided to study the genetics and molecular mechanism during interaction with these pathogens. A protocol for the infection of *Brachypodium distachyon* with *Magnaporthe grisea* (rice blast) is developed to study the dynamic host/pathogen interactions^21^.When inoculated with *Fusarium* head blight (FHB) isolates, *Fusarium graminearum* and *Fusarium culmorum*, *Brachypodium distachyon* exhibits characteristics of susceptibility highly similar to those of wheat and barley, including susceptibility to spread of disease in the spikelets^22^, and two clusters of *Brachypodium distachyon* UDP-glycosyltransferases could detoxify the mycotoxin deoxynivalenol produced by FHB, conferring the resistance to FHB^23^.The compatible interaction of two *Brachypodium distachyon* accessions with eyespot and ramularia leaf spot causal agents, *Oculimacula* spp and *Ramularia collocygni* is tested, and *Brachypodium distachyon* accessions develop symptoms similar to those on the natural host^24^. *Brachypodium distachyon* is a resistant nonhost to *Zymoseptoria tritici*, the causative organism of wheat *Septoria tritici* blotch disease, with the variant resistance responses ranging from immunity to a chlorotic/necrotic phenotype^25^. The disease development has been evaluated to assess the utility of the *Puccinia graminis*-*Brachypodium* pathosystem to investigate the molecular and genetic basis of stem rust resistance, and demonstrated the variation in stem rust resistance, from partially susceptible to almost immune^26^. Up to 140 *Brachypodium distachyon* accessions were infected with selected cereal rust, including *Puccinia graminis* f. sp. *tritici*, *Puccinia triticina*, and *Puccinia striiformis*, *Puccinia* graminis ff. spp. *avena* and *phalaridis*, and related *Brachypodium distachyon* lines show similar cytological symptoms to their host^27^.

To date, large collections of *Brachypodium distachyon* T-DNA insertion mutant lines has been established, such as the available 23, 000 lines at Joint Genome Institute (JGI), and 13, 000 lines produced by the BrachyTAG programme and USDA-ARS Western Regional Research Center (in the year 2010). These collections are mainly used to do the research about the biomass quality and agronomic characteristics of cereal and energy crops. Here, the generated *Brachypodium distachyon* T-DNA insertion population focused on the mechanism of durable resistance to wheat stripe rust. Here our study explored the potential of *Brachypodium distachyon* T-DNA insertion lines as nonhost and their ability to serve as model pathosytem to study NHR to wheat stripe rust.

## Results

### The typical NHR microscopic interaction of Bd21 and CYR32

**Figure 1:**
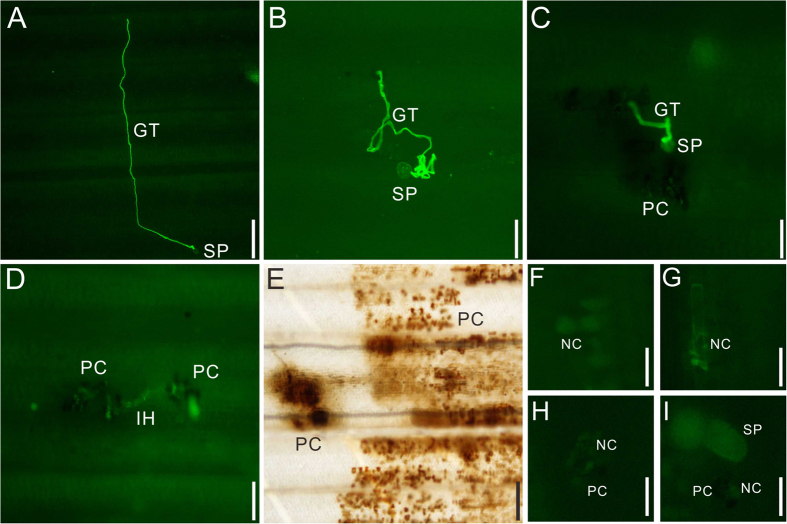
The microscopic observation of wheat stripe development in Bd21. (**A**,**B**) The long and zigzag germ tube of wheat stripe rust. (**C–E**) The black spots show the numerous pigmented cells around the infection site. (**F**,**G**) The necrotic cells that have autofluorescence appeared in the infection site and around the PC area. SP, spore. GT, germ tube. IH, infection hypha. PC, pigmented cells. NC, necrotic cells. (**A**,**E**) bar = 100 μm, (**B–D**,**F**,**G**) bar = 5 μm, (**H**,**I**) bar = 2 μm.

We conducted microscopic study of stripe rust development in Bd21 wild type, and the mutants’ population that includes resistant and susceptible. The infection behavior of CYR32 in Bd21 was somehow similar to that in partially resistant wheat cultivars. Microscopically, almost all the spores (SP) could germinate in the leaves of Bd21, but only a few spores could successfully complete the entire infection process. And for most spores, several key stages of infection process were inhibited. In Bd21, the germ tube (GT) of the germinated spore penetrated into distant stoma after going through a long or zigzag way and escaping the nearby stoma (Fig. 1A,B). The necrotic patches were noted around the stomata and pigmented cells (PC) showing the programmed cell death or hypersensitive response (HR) of the host to hinder the further invasion by the germ tube or penetration into stomata in Bd21 (Fig. 1C,F,G and I). Meanwhile, this kind of HR occurred during the process after the establishment of infection site, such as the formation of the substomatal vesicles (SV) and haustorium (Fig. 1C,I), and the expansion of the infection hypha (IH) (Fig. 1D,E and H).

Among the mutants, the resistant lines responded to PST by HR and inhibition of pathogen was observed at different stages. We recorded similar response for Bd21 as described above. So here the infection process of PST in resistant mutants was not shown.

### The successful infection process in susceptible T-DNA insertion mutants

After the large scale screen to the generated T-DNA insertion population with CYR32, hundreds of confirmed mutants associated with different pathologic phenotypes were identified. And the infection process of spores of stripe rust pathogen in susceptible mutants was observed.

**Figure 2:**
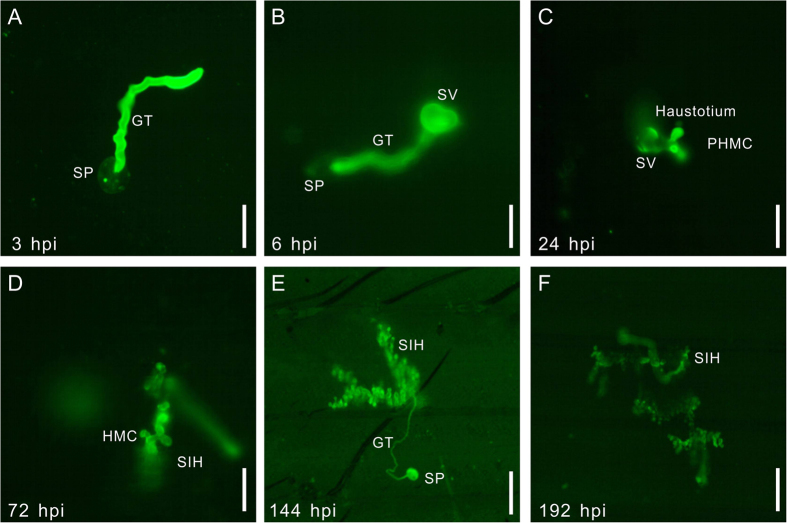
The microscopic observation of wheat stripe rust development in the susceptible T-DNA insertion mutants. (**A**) At 3 hpi (hours post inoculation), the germ tube (GT) develops from the germinated spore (SP). (**B**) The GT enter the stomata and substomata vesicle (SV) formed at 6 hpi. (**C**) The haustorium develops from primary houstorium mother cell (PHMC) at 24 hpi. (**D**,**E**) The secondary infection hypha (SIH) expand among the leaf cells. (**A**–**D**) bar = 2 μm, (**E**–**F**) bar = 5 μm.

The infection process of wheat stripe rust in susceptible mutant lines was similar to susceptible wheat cultivars. Among the susceptible mutants almost all the spores after germination could complete their infection process. The development time and fungal mass of PST in susceptible mutants varied slightly, while the infection behavior of PST remained similar, including the germination of spore, entrance of germ tube to the stoma and extension of hypha in the leaf surface. So here we reported the infection process of PST in one susceptible mutant as a representative infection behavior of the susceptible mutants. Upon inoculation of the leaves, the spores developed germ tube at 3 hpi (hour post inoculation) to elongate towards stomata over the leaf surface (Fig. 2A). Later, the GT entered the leaf through the stomata and the SV formed at 6 hpi, which was a symbol of the establishment of infection site (Fig. 2B). Then the SV produced the primary haustorial mother cells (PHMC), from which the haustorium developed at 24 hpi (Fig. 2C). This primary infection hypha grew along the gap of the leaf cell with the possible nutrition absorption from the leaf cells by the haustorium (Fig. 2D), and the area of the infection hypha became larger (Fig. 2E,F), and at about 14 dpi (days after inoculation), the infection hypha began to break through the leaf surface and urediniospores appeared gradually. And the final disease phenotype in *Brachypodium distachyon* to PST was observed at about 20–30 dpi.

### The phenotypic responses of Bd21 and T-DNA insertion mutants to CYR32

**Figure 3:**
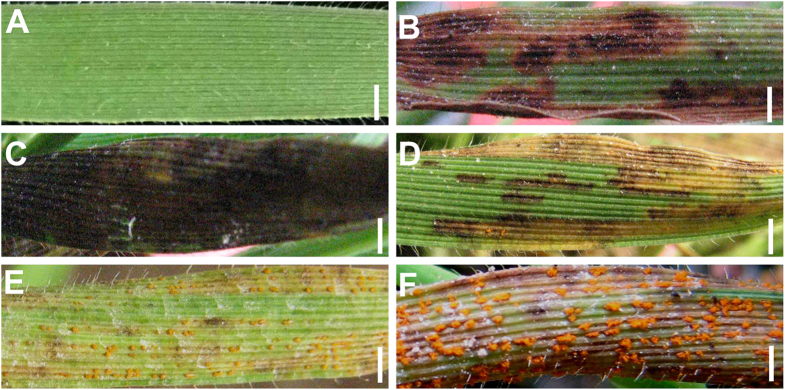
The grades of different pathologic phenotype. (**A**) The immunity phenotype, 0 infection type. (**B**,**C**) Necrotic spots without spore, black and brown spot, 1–2 infection type. (**D**) Bd21-like phenotype, necrotic area with trace/light sporulation, 3–4 infection type. (**E**) Moderately susceptible, moderate sporulation with/without necrotic area 5–7 infection type. (**F**) Highly susceptible, abundant sporulation, 8–9 infection type. Bar = 1.5 mm.

The phenotypic assessment of Bd21 under stripe rust disease conditions revealed small to medium and large necrotic patches on the leaf surface, with countable spores only in the margin and tip of leaf, which was the typical NHR (Fig. 3D). In the susceptible T-DNA insertion mutants, the leaf was covered by spores, while in the resistant T-DNA insertion mutants, the spores could also germinate, but the infection process was totally inhibited by HR, and the final symptoms were black or brown flecks. Also, some mutants did not show any obvious phenotype before and after the inoculation, thus expressing total immunity to CYR32.

Mutants that had the similar phenotype with Bd21 were treated as the same grade Bd21 (Bd21-like). Combined our visible phenotype pictures (Fig. 3A–F) and the 0–9 infection types of wheat stripe rust^28^, we divided the mutants into five groups to evaluate the visible disease phenotype (shown in Table 1)

In the 7,000 T-DNA insertion mutants’ collection, the number of immunity (0 infection type) mutants is 24, and the number of resistant (1–2 infection type) mutants is 28, and the numbers of the moderate susceptible (5–7 infection type) and highly susceptible (8–9 infection type) mutants are 89 and 66, respectively. Supplemental Table 1 shows the list of main classification of some typical mutants. The T-DNA flanking sequences of many lines have been derived and was presented in Supplemental Table 2.

### T-DNA mutants with increased susceptible and resistant phenotype

Based on morphological and pathological evaluation, we categorized mutants into two groups: the mutants that had both pathologic and morphologic phenotype and the others had only pathologic phenotype. The first group included one mutant having morphological and pathological phenotype T2638. The other group included two mutants having pathological phenotypes of susceptibility and resistance, T40 and T719. The flanking sequences of the three mutants could be derived very easily by the first time IPCR, and the T-DNA insertion was single in these three mutants from genetic analysis of a large population (T719 in a small population, about 20 individuals).

**Figure 4:**
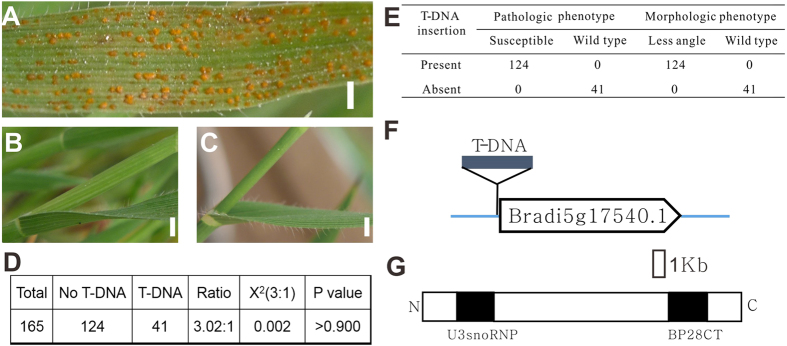
The pathologic phenotype and primary genetic analysis of mutant T2638. (**A**) The susceptible line T2638. (**B**) The less angle between petiole and stem than Bd21 (**C**). (**D**,**E**) The genetic analysis of T3 generation of T2638. (**F**) The T-DNA insertion site of T2638 in the gene *Bradi5g17540*. (**G**) The two conserved domains in the protein of *Bradi5g17540.* (**A**) Bar = 1.5 mm. (**B**,**C**) Bar = 3 mm.

Compared to wild type Bd21 (Fig. 3D), the mutant, T2638, showed increased susceptibility to CYR32 (Fig. 4A). Besides the pathologic phenotype, this mutant showed less angle between petiole and stem than the wild type (Fig. 4B,C). Among the 165 T3 offspring plants, 41 lacked the T-NDA insertion, and 124 had the T-DNA insertion (Fig. 4D), and the ratio was 1:3. All plants without the T-DNA insertion showed the same pathologic and morphologic phenotype as Bd21, and all plants with the T-DNA insertion showed susceptibility to CYR32 and less angle between petiole and stem (Fig. 4E). And this above mutant contained a T-DNA insertion in the promoter of the gene *Bradi5g17540*, just about 240 base pairs preceding the start codon ATG (Fig. 4F). These results suggested that *Bradi5g17540* might be a putative candidate gene that caused the morphologic and pathologic phenotype of T2638. *Bradi5g17540* encoded an uncharacterized *At3g06530*-like BAP28 protein. It contained two conserved domains, U3snoRNP domain in the N terminal and BP28CT domain in the C terminal (Fig. 4G). Further study containing complementary experiments are required to confirm the interaction between the gene *Bradi5g17540* and the phenotype of T2638 in the future.

**Figure 5:**
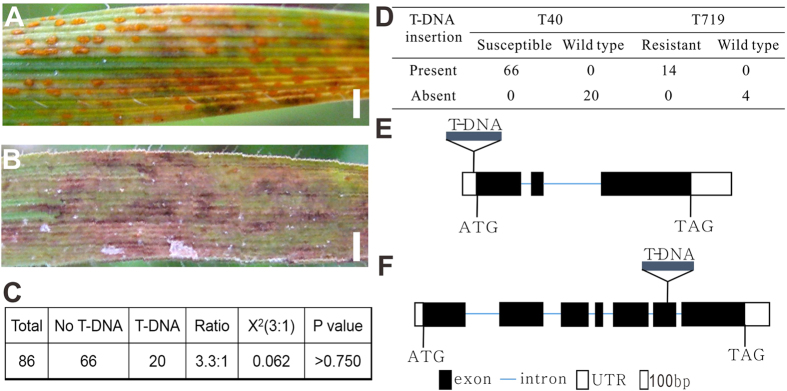
The pathologic phenotype and primary genetic analysis of T40 and T719. (**A**) The susceptible line T40. (**B**) The resistant line T719 compared to Bd21. (**C**) The genetic analysis of T3 generation of T40. (**D**) Co-segregation of the T-DNA insertion with the phenotype of T40 and T719. (**E**) The T-DNA insertion site of T40 in the gene *Bradi5g15760*. (**F**) The T-DNA insertion site of T719 in the gene *Bradi5g11590*. Bar = 1.5 mm.

The second mutant, T40, also showed increased susceptibility responding to CYR32 compared to wild type Bd21 (Fig. 5A). Among the 86 T3 offspring plants, 20 lacked the T-DNA insertion, and 66 had the T-DNA insertion, and the ratio was about 1:3(Fig. 5C). All plants without the T-DNA insertion showed the same phenotype as Bd21 to CYR32, and all plants with the T-DNA insertion showed increased susceptibility (Fig. 5D). And the T-DNA inserted in the 5′ UTR of the gene *Bradi5g15760*, which encodes a MYB family transcription factor, *BdMYB102* (Fig. 5E). And these primary results suggested that the increased susceptible phenotype of T40 was likely caused by the T-DNA insertion in *BdMYB102.* MYB proteins are key factors in regulatory networks controlling the development, metabolism and responses to biotic and abiotic stresses. Studies in *Arabidopsis*, rice, wheat and tabacoo show that the many members of MYB transcription factor are involved in defense to bacteria, fungi and virus pathogens^29^,^30^,^31^,^32^. In this mutant T40, the expression level of *BdMYB102* was slightly changed by the inserted T-DNA (data not shown), and might have caused the failure of active plant defense to CYR32, and resultantly showed the increased susceptible phenotype.

A third mutant, T719, was resistant to the wheat stripe rust, with large area of brown necrotic areas patches resulting from the hypersensitive reaction of cell death (Fig. 5B). And an identified inserted gene was *Bradi5g11590*, which was found to be a lipoxygenase (LOX) (Fig. 5F). LOXs are a family of iron-containing enzymes that catalyze the dioxygenation of polyunsaturated fatty acids in lipids, and previous studies have shown that the LOX-pathways are involved in regulating defense and programmed cell death responses to microbial pathogens^33^,^34^. Therefore, the LOX gene *Bradi5g11590* could be a putative candidate gene responsible for the resistant phenotype of T719.

## Discussion

The large size genome and numerous highly repetitive sequences severely hindered the cloning of wheat resistant genes and the research on molecular mechanism underlying the interaction with pathogens. The classic model plant *Arabidopsis*, was not compatible to the wheat stripe rust^35^, and in spite of nonhost resistance, it could not serve as a model nonhost because of taxonomic distance and genetic barriers. Here, the *Brachypodium distachyon* was a nonhost to the wheat stripe rust based on phenotypic response to stripe rust, and it was convenient to get susceptible and resistant mutants. Therefore, *Brachypodium distachyon* will provide an optimal system to study the molecular mechanism of NHR, the durable resistance to wheat stripe rust.

In wheat, the major effective *R* genes were used in most wheat cultivars for controlling wheat stripe rust. However, lacking of durability of *R* genes often results in a periodic disease pandemic that threatens food security. Exploration of molecular mechanism of NHR and other durable disease resistances to wheat stripe rust would be in favor of broadening our ideas for breeding durably resistant cultivars. Use of the genetically related species *Brachypodium distachyon* to study the NHR to wheat stripe rust would allow us to more easily dissect the molecular mechanism of durable resistance, and that would provide a theoretical basis for breeding wheat cultivars that are more durably resistant to wheat stripe rust.

Previous studies in the last decade have been focusing on the the cloning of genes invloved in the NHR, and identified many genes participating in this kind of resistance. *NHO1* is the initially found gene conferring *Arabidopsis* the ressitance to P. *syringae* pv. *Phaseolicola*^36^, and later the suppressor of the G2 allele of S-phase kinase-associated protein 1 SGT1^37^, WRKY46 and WRKY54^38^, squalene synthase SQS^39^, glycolate oxidase GOX^40^ and many other genes are identified in succession to be inovlved in the NHR. The three candidate genes identified here are likely to be responsible for the increased susceptible and resistant phenotype in the T-DNA insertion mutants. *BdMYB102* is a transcription factor, like the WRKY46 and WRKY54, and *Bradi5g11590* is a LOX enzyme, and SQS and GOX are also enzymes. *Bradi5g17540* is a BAP28 domain containing protein, and this domain is found to be required for cell survival in the central nervous system in animals^41^. Therefore, the three genes may participate in the nonhost interaction between *Brachypodium distachyon* and wheat stripe rust.

The main purpose of the T-DNA insertion population established here is to find the mutants associated with pathology, and at the same time, during our screening process, hundreds of mutants showed abnormal morphologic phenotype compared to Bd21. The major phenotype of most of these mutants can be assigned to two categories, dwarfism and curl leaf. The proportion of dwarf ones is very large, and the dwarfism level is variant. The number of curly-leaf mutants is about twenty-five, and the curly index is also different. There are more downward-curly mutants than upward-curly ones. These two kinds of mutants can be employed to study the establishment of plant architecture. Besides these two main morphologic mutants, there are countable mutants showing other abnormal phenotype, such as prostrate tillers and defective flowers. The collection of these mutants will be a powerful resource for systematic elucidation of gene function in grass species. What is more, some mutants not only show pathologic symptoms to CYR32, but also display abnormal morphologic phenotype, and studies on such mutants will present the tradeoff between plant development and defense.

The flanking sequences listed here are just the sequences that we could derive using IPCR, but there may be other flanking sequences for some certain T-DNA mutants, which denotes some mutants may have more than one T-DNA insertion copies. We could acquire two or three flanking sequences in a single IPCR, but not all the T-DNA flanking sequences could be derived by this method. The possible reason may be the restriction of IPCR itself, or the genome microenvironment around the T-DNA insertion site and the insertion manner of T-DNA^42^. And all the flanking sequences may be derived by combination of IPCR and other methods, such as thermal asymmetric interlaced PCR (TAIL PCR) and adaptor-mediated PCR. Therefore, for a single desirable mutant that have many T-DNA insertion copies, genetic analysis should first be committed to find the phenotype linked T-DNA insertion, and then we can use southern blot or real-time PCR to verify the T-DNA copy number, and next clone the flanking sequences we want.

The subsequent genetic analysis showed that the phenotype of some mutants is not linked to the identified T-DNA insertion site, and it may be caused by other T-DNA insertion or the tissue culture. Even for the three mutants we showed here, it is possible that other gene(s) or T-DNA insertion site(s) may contribute to the pathogenic phenotype, and more detailed confirmation or experiments are needed to identify the real responsible gene(s) for the selected mutant in the future. It is likely that the responsible gene of T-DNA insertion mutant may be not caused by the gene nearest to the T-DNA insertion^43^,^44^,^45^. However, the T-DNA tagged collection here still is a viable mutant population to study NHR, since the T-DNA insertion mutant lines have the different pathologic phenotype with the wild type, we can use these mutants to study the molecular mechanism of NHR to wheat stripe rust.

## Methods

### Generation of T-DNA insertion Bd21 lines

The T-DNA insertion Bd21 lines were generated through *Agrobacterium*-mediated transformation of embryogenic calli of Bd21 immature embryos according to the method provided by Vain^46^. The binary vector used here was pK7G2D, a gateway system, which contained a GFP marker driven by CaMV35 promoter, and as a result of successful transformation the positive mutants were visible under the UV light indicating the presence of marker. The *Agrobacterium* strain EHA105 was employed to initiate the transformation.

### Retrieval and analysis of T-DNA flanking sequences

The sequences flanking the T-DNA inserts were retrieved using an inverse PCR (IPCR). Briefly, genomic DNA was extracted from T0 plants using CTAB method. The DNA was digested by the restriction endonuclease ApaI for about 2 hours, and the digested fragment was purified by high speed centrifuge. And then T4 DNA ligase system was added to the DNA precipitation to induce self-ligation. Nested primers specific to the left border were used in two rounds of PCR to amplify the flanking sequences. The sequence alignment was committed in the main web phytozome (http://www.phytozome.net/) and *Brachypodium* at PlantGDB (http://www.plantgdb.org/BdGDB/).

### Plant and fungal material

CYR32, a wheat stripe rust isolate, has been the most dominant race with the highest frequency in the majority of wheat growing areas of China and played a major role in causing the widespread epidemics of wheat stripe rust in the past several years^47^. So the wheat stripe rust isolate, CYR32, was used in this study. The wheat cultivar, Mingxian 169 highly susceptible to CYR32, was used to propagate the spores. Mingxian 169 was grown to three-leaf stage in a soil/vermiculite (1:1) mixture at 20°C for 16 h of light and 8 h of darkness in a culture room. For CYR32 inoculation, the seedlings were transferred to 12 °C in a humid plastic greenhouse.

The *Brachypodium distachyon* T-DNA insertion population was generated from Bd21, and the plants were grown at 14 ± 3 °C for 20 h of light and 4 h of darkness in a temperature controlled plastic greenhouse supplemented with additional lighting.

### Propagation of wheat stripe rust spores

The spores of wheat stripe rust, CYR32, stored at −80 °C in liquid nitrogen were kept moist with sterile water in 4 °C for 12 h. When the second leaf of Mingxian 169 expanded fully, the surface of the leaf was sprayed uniformly with 0.02% (v/v) Tween 20. Then the small water droplet covered leaves were inoculated by CYR32, diluted 20 folds with talcum powder in the inoculation tower. Then the inoculated seedling of the wheat were kept wet under dark for 24 h. After about 20 days, the spores were collected from the infected wheat leaves.

### Inoculation of *Brachypodium distachyon*

The inoculation of *Brachypodium distachyon* was committed in plastic green house in winter. In brief, the leaves of Bd21 T-DNA insertion mutants were sprayed with 0.02% (v/v) Tween 20 using an agricultural sprayer, and then the talcum powder diluted fresh spores were sprayed uniformly to the surface of plant leaves with a larynx atomizer. The inoculated plants were kept wet under dark for 24 h.

### Assessment of disease phenotype

The disease phenotype assessment was done according to the final visible symptoms developed in the infected leaves compared to the wild type. And the classic 0–9 infection type of wheat stripe rust^28^ was employed to evaluate the disease phenotype.

### Leaf sample preparation and histopathological analysis

We used the adapted method reported by Zhang *et al*^31^. (2012). Leaf segments were cut from the inoculated leaves, fixed and decolorized in ethanol/trichloromethane (3:1, v/v) containing 0.15% (w/v) trichloroacetic acid for at least 2 days, and replaced the buffer once. The specimens were cleared in saturated chloral hydrate until leaf tissues became semi-transparent (2–3 days). Then the HR could be observed under Nikon ECLIPSE 80i microscope.

For WGA-Alexa 488 staining, after rinsed twice with sterile water, the specimens were soaked in 1 M KOH three times for 10 min. Then samples were incubated in 50 mM Tris-HCl pH 7.5 for 20 min. A majority of the Tris-HCl solution was then removed and a 1 mg/ml solution of WGA-Alexa 488 was added to a final concentration of 20 μg/ml staining solution. After staining, the buffer was removed from the tube, and the tissue was rinsed in sterile water twice. Specimens were stored in 25% (v/v) glycerol. All WGA-Alexa stained tissues were examined under Nikon ECLIPSE 80i microscope.

## Additional Information

**How to cite this article**: An, T. *et al. Brachypodium distachyon* T-DNA insertion lines: a model pathosystem to study nonhost resistance to wheat stripe rust. *Sci. Rep.* **6**, 25510; doi: 10.1038/srep25510 (2016).

## Supplementary Material

Supplementary Information

## Figures and Tables

**Table 1:** The phenotypic categories of the mutant’s lines.

Groups	Infection type	Phenotype
Immunity	0	No visible phenotype
Resistance	1–2	Necrotic area
Bd21-like	3–4	Necrotic area with trace/light sporulation
Moderately susceptible	5–7	Moderate sporulation with/without necrotic area
Highly susceptible	8–9	Abundant sporulation
