# Supplementary material for: Brachypodium distachyon T-DNA insertion lines: a model pathosystem to study nonhost resistance to wheat stripe rust
Source: Sci Rep. 2016 May 3;6:25510. doi: 10.1038/srep25510 (PMC4853781; doi:10.1038/srep25510)
Supplement: Supplementary Information [file srep25510-s1.pdf]

Brachypodium distachyon T-DNA insertion lines: a model pathosystem to study nonhost resistance to wheat stripe rust

Tianyue An, Yanli Cai, Suzhen Zhao, Jianghong Zhou, Bo Song, Hadi Bux ＆ Xiaoquan Qi*

1 List of different pathologic phenotype of T-DNA insertion mutants*

Infection type	Group	Mutants list					
0*	Immunity	343	802	987	1123	1174	1289
		1293	1384	1402	1587	1877	1923
		2134	2244	2567	2732	2841	2901
		3343	3846	4024	4131	4824	4936
1-2	Resistant	719	988	1294	1705	2739	3847
		3920	3965	4033	4253	4236	4261
		4294	4339	4424	4513	4540	4561
		4583	4597	4606	4660	4662	4720
		4711	4761	4768	5005		
5-7	Moderate susceptible	406	544	585	904	1173	1330
		1763	2121	2518	2522	2524	2560
		2583	2643	2668	2698	2707	2714
		2757	2763	2781	2797	2815	2817
		2819	2858	3087	3334	3432	3437
		3526	3801	3975	4021	4073	4140
		4187	4209	4357	4360	4412	4429
		4468	4594	4621	4619	4652	4659
		4661	4672	4762	4829	4942	4996
		5067	5037	5093	5117	5138	5168
		5174	5200	5220	5303	5385	5993
		6031	6068	6153	6160	6187	6228
		6240	6248	6250	6291	6328	6332
		6352	6366	6380	6393	6400	6452
		6457	6468	6469	6483	6829	
8-9	Highly susceptible	22	40	213	661	1427	1903
		1978	1988	2537	2607	2638	2655
		2658	2662	2725	2727	2738	2740
		2939	3051	3158	3162	3303	3399
		3456	3460	3824	3836	3843	3865
		3878	3889	3894	3903	3931	3939
		3951	3998	4027	4051	4091	4154
		4191	4200	4415	4474	4618	4663
		4679	4724	5042	5102	5454	5476
		5547	5786	5807	5846	5892	5906
		5942	5949	5952	5969	6169	6663

*: Some lines of the 0 scale may be due to the escaping the inoculation. To obtain T-DNA mutants, please contact the corresponding author.

2 The T-DNA left border flanking sequence of 24 T-DNA insertion lines (5'-3')

>T22

CTGTGGGGTCGGATCTAGCTAGAGTCAGCAGATCGTTCAAACATTTGGCAATAAAGTTTCTTAAG

ATTGAATCCTGTTGCCGGTCTTGCGATGATTATCATATAATTTCTGTTGAATTACGTTAAGCATG

TAATAATTAACATGTAATGCATGACGTTATTTATGAGATGGGTTTTTATGATTAGAGTCCCGCAA

TTATACATTTAATACGCGATAGAAAACAAAATATAGCGCGCAAACTAGGATAAATTATCGCGCGC

GGTGTCATCTATGTTACTAGATCGACCGGCATGCAAGCTGATAATTCAATTCGGCGTTAATTCAG

TACATTAAAAACGTCCGCAATGTGTTATTAAGTTGTCTAAGCGTCAATTTGTTTACACCACAATA

TATCCAAAATTGAATCCGGTATCTGTCTATCTATCAACAACTAATAAAGCTTTCTTCAATCATCT

CCGTGCCCTATTAGCATCGCAGATAATAAGGGAAAAAAAAATCTCTCCTCAAGTTCGGGGTGCTT

ACCGTGAGCTCTCCCATATGGTCGACTAGAGCCAAGCTGATCTCCCTTTGCCCA

>T40

GCGGGTCGTCGGTCTTGACAAAGACCGGGCGCCCCTGCGCTGACAGCCGGAACACGGCGGCATCA

GAGCAGCCGATTGTCTGTTGTGCCCAGTCATAGCCGAATAGCCTCTCCACCCAAGCGGCCGGAGA

ACCTGCGTGCAATCCATCTTGTTCAATCATGCCTCGATCGAGTTGAGAGTGAATATGAGACTCTA

ATTGGATACCGAGGGGAATTTATGGAACGTCAGTGGAGCATTTTTGACAAGAAATATTTGCTAGC

TGATAGTGACCTTAGGCGACTTTTGAACGCGCAATAATGGTTTCTGACGTATGTGCTTAGCTCAT

TAAACTCCAGAAACCCGCGGCTCAGTGGCTCCTTCAACGTTGCGGTTCTGTCAGTTCCAAACGTA

AAACGGCTTGTCCCGCGTCATCGGCGGGGGTCATAACGTGACTCCCTTAATTCTCCGCTCATGAT

CGATAATTCGCGGTACCCGGGGATCCTCTAGAGGGCCCATGGAGATAACATATTGTATGCCCCCC

ACAAGCAAATTGACGCTTAGACAACTTAATAACACATTGCGGACGTTTTTAATGTACTGAATTAA

CGCCGAATTGAATTATCAGCTTGCATGCCGGTCGATCTAGTAACATAGATGACACCGCGCGCGAT

AATTTATCCTAGTTTGCGCGCTATATTTTGTTTTCTATCGCGTATTAAATGTATAATTGCGGGAC

TCTAATCATAAAAACCCATCTCATAAATAACGTCATGCATTACATGTTAATTATTACATGCTTAA

CGTAATTCAACAGAAATTATATGATAATCATCGCAAGACCGGCAACAGGATTCAATCTTAAGAAA

CTTTATTGCCAAATGTTTGAACGATCTGCTTGACTCTAGCTAGATCG

>T406

TTCTTTTTTGGGGGGCGTCCTCCTCTTATTAGGCTCCGACACATCGTTCAACTATTTGCGGACGC

CTTTGTGCTGAATTTCCCCTGCTGAGTCTTCCGACAAACCAATCAAAAATCTCTCTCATTGCCTG

ACCACCTCTACATTGCGGAGTAAGATCCTGCGGCCGCTTTTTTGCACAACATGGGGTATCATGTA

ACTCCCCTTGATCGTTGTTAACCGGAGCCATTCTACCGACGCAACACCAGCAACATGCGGTAGCT

CTGCAGTATTGGGAACTCACTGGCCGTCGTTTTACCCGTCGTGACTGGGAAAACCCTGGCGTTAC

CCAACTTAATCGCCTTGCAGCACATCCCCCTTTCTCAGCTGGCGTAATAGCGAAGAGGCCCGCAC

CGATCGCCCTTCCCAACAGTTGCGCACCCTGAATGGCGAATGGACGCGCCCTGTATCGGCACATT

AAGCGCGGCGGGTGTGGTGGTTACGCGCATCGTGACCGCTACACTTGCCAGCGCCCTAGCGCCCG

CTCCTTTCGCTTTCTTCCCTTCCTTTCACACCACGTTCGCCGGCTTTCCCCGTCAAGCTCTAAAT

CGCGGGCTCCCTTTTGGGTTCAGATTAAGTGCTTTACGGCACCTCGACCCCAAAAAACTTGATTA

GGGTGATGGTTCACGTAGTGAGCCATCGACCCTGATAGACGGTTTTTCTCCCCTTTGACTAGCCA

GGCTGACTATGGGATGAACGAATACACAGATCGCT

>T544

CAGCCTCGTTCGATTGAGAGTGATATGAGACTCTAATTGGTACCGAGGGGATTTATGGAACGTCA

GTGGAGCATTTTTGACAAGAAATATTTATTTATGAAAGTCAATAACTCAACATGCTTTCTCTCCA

ACAGAAGTCCTTGCTGCCAGTGGACGGCAACCAATCAGAAATGACCAAAAACATGTCAGATACAT

GCAGGCCAGCCTTGTTCCAGTCTTTGGCAATTTGGGACTAAATAAAGCCTCTGGCTTAACCTCAT

GCTGTTGTAGCTGTTGCACCAAGATCATTCAGTTAGGCAAAATTCTGTTTGTTGCTATGTGCCAT

TAACTCATTGGTGGTTGCAATTAAAGAATGTTGCAACTTCTCAGGGGAGGAGGGGGGTCACCGGT

CACTGAGTCACATGCGCCGGAAAATGCATAGGACCGAACAAGCAGCTGGAATCTCTGCCACAGCT

CACACGCGCTGTTGGACAAAACGGTAGGTATATGACTAGGACTTTGCCATTCCAGCTACCTAAAA

ACTGGAACTGTATTCTCTCCGTATTTTTTTCTATTCCGTGATCACATCAACATGAAGATTTTTTT

TATAGCGTCAATCTTTTTCCTTTCTATTCTTTCAATGATTGGCTCCAGGACACAGATTGTGCTTA

ATCCAAATGCTGATTCTGAAATGTGAACAACTGGGTCTTTTGGCAACTTTCAGTGTGAAAACAAT

ATCTTTCAGTGCATAAAAATATGTTAGGAAAAACAATCTTCTTGCCTGTTTGTGTTGCAAAAACT

GAGCGAGAGGGACCATAGAGATCCATCCATCACCCATCAATTAGTAATAGCCTAATATGAGATCA

GGACAGGCACCTTTCGAAGAGCATGATACCTGTTCTGCCACTGTGACACGCACCATCCCGGAAGC

AACTTCCTGCCGAAGCAGCTTTCTTAGGCTGACAAGCAACTGTATGCGTAGATTAATCATAATCG

TGTGGCTGCCGACTGCGTCAAGCATTCCGCGAATCTAGGTAAGGTCCTCGGTTACACTCGAATCG

ACTTTCTTTTAACTTGAAGACACATTCATGTAAAGCGGTTTGTACTATGTACTTACTCCGCTGAC

TACTAGTACTCGGTGTCATGCTTGAAAGAGAAAGTCAACTATTCTCGTATGACAAT

>T661

CCGAACGCGCTCGATTCGCAGCGCATCGCCTTCTATCGCCTTCTTGACGAGTTCTTCTGAGCGGG

ACTCTGGGGTTCGGACTCTAGCTAGAGTCAAGCAGATCGTTCAAACATTTGGCAATAAAGTTTCT

TAAGATTGAATCCTGTTGCCGGTCTTGCGATGATTATCATATAATTTCTGTTGAATTACGTTAAG

CATGTAATAATTAACATGTAATGCATGACGTTATTTATGAGATGGGTTTTTATGATTAGAGTCCC

GCAATTATACATTTAATACGCGATAGAAAACAAAATATAGCGCGCAAACTAGGATAAATTATCGC

GCGCGGTGTCATCTATGTTACTAGATCGACCGGCATGCAAGCTGATAATTCAATTCGGCGTTAAT

TCAGTACATTAAAAACGTCCGCAATGTGTTATTAAGTTGTCTAAGCGTCAATTTGTTTACACCAC

AATATCGAAGCGATGAGCAATGCCATGGGTCATCATACGCGGTATGACGAGGGATGACCTGCACT

GGTCTATGTGAAGTTTTTATGCTCACTATCAATTTAAACAACTGTCAGGCCCGCACGATGTTAAT

TAATTAATGTACCCGACCTAGAATTACCGCCTCGGATTGCTCGAGTAGATCGATAGATTCGCGTC

GCCAAACACCGGTCCCGCACGCGACGCACCACACCACGAACGACGAACCCCCACCGCTTTTCTGC

CGAGCACGGAACCCCCAAAACCCCTAAATTCCCCACCCCGATCGCCTCCCCTGCCGCCGATCTCG

CCGGAACCCCAGCAGGTCTCTCCGCCCGGCCGCGATGGATTCCGATGATGATCTGCGGGTCGCCA

GCGACTCGGAGCTGGTCGACGGCGCGGACTACCACTACTGCAGCGACGGGGAGAGCAACGGCAGC

AGAGCGACGACGACGGTTCGACGTTGGCGGCGACGCCTACGAGGTCGGCGACGAGGTCGTCGCGA

TGCGCG

>T719:

CCATCGGTTTGGATCTAGCTAGAGTCAGCAGATCGTTCAAACATTTGGCAATAAAGTTTCTTAAG

ATTGAATCCTGTTGCCGGTCTTGCGATGATTATCATATAATTTCTGTTGAATTACGTTAAGCATG

TAATAATTAACATGTAATGCATGACGTTATTTATGAGATGGGTTTTTATGATTAGAGTCCCGCAA

TTATACATTTAATACGCGATAGAAAACAAAATATAGCGCGCAAACTAGGATAAATTATCGCGCGC

GGTGTCATCTATGTTACTAGATCGACCGGCATGCAAGCTGATAATTCAATTCGGCGTTAATTCAG

TACATTAAAAACGTCCGCAATGTGTTATTAAGTTGTCTAAGCGTCAATTCGTTGCTGATGAAGTC

TGCAAACGAGTGAGATCAGCAGAGACATCTGGGCATTGTACGTACCTTCTGATGAGGTCGGCGGG

GAGGCCTTCCTGGTCGAGCCGCCAGAGGTTGTCGTAGGCGAAGGAGCTCTCCCATATGGTCGACT

AGAGCCAAGCTGATCCCCTTTGCCCAGGCCGTGCACTCCGACTACGAGCTCTCCCATATGGTCGA

CTAGAGCCAAGCTGATCTCCTTTTGCCCCCCAA

>T988

CGGACGCGCTCGATTCGCAGCGCATCGCCTTCTATCGCCTTCTTGACGAGTTCTTCTGAGCGGGA

CTCTGGGGTTCGGACTCTAGCTAGAGTCAAGCAGATCGTTCAAACATTTGGCAATAAAGTTTCTT

AAGATTGAATCCTGTTGCCGGTCTTGCGATGATTATCATATAATTTCTGTTGAATTACGTTAAGC

ATGTAATAATTAACATGTAATGCATGACGTTATTTATGAGATGGGTTTTTATGATTAGAGTCCCG

CAATTATACATTTAATACGCGATAGAAAACAAAATATAGCGCGCAAACTAGGATAAATTATCGCG

CGCGGTGTCATCTATGTTACTAGATCGACCGGCATGCAAGCTGATAATTCAATTCGGCGTTAATT

CAGTACATTAAAAACGTCCGCAATGTGTTATTAAGTTGTCTAAGCTGTGTCTTCTCCCTCAATTT

ATGTTGCATTGCTGAGTTGCTGTCAGCTCCTCGGCCTCTTTCCAATCCCAACCCCGGCAGGCAGG

AGATAGGCGTGAGGAGCGAGGATGGCGAGTCCGCAGTGCTGCGCGAACCCGCCGACGCTGAACCC

CGCCGGCGGAGAGGGCAAGGTCGTCGACAGCTTCGGCGGGACCAGGGCGTACGTCGCCGGCGCTG

AAGAGTCCAAGGCCGCCGTCATCCTCATCTCCGACATCTTCGGTGCTTGGTTTTGAATCCCCTCC

TCGGTATCTCATGTAGTCCTCCCAAATGCATTGCTCTGCTCGGCATAGTAACAACAACCACTTTC

TGTGTCCTCCTTGCAGGGTTTGAAGCGCCGAAACTGACGTATACAATTTTGTGCTCTCTGATATC

ACGAATTCGTATTTGCTTCGTCATGTTTTGGTTTAGCAATGACCTATGTGTCGGAGGCAGGATGG

TGTGGTTGTTAACTGTTTCGGGGGATGTGGTATTAGAAACAATGATGATGTGGTGTATAGTATGC

GATGCT

>T1173

AAAGGCCCTCGTTCGAGTTTGAGAGTGATATGAGACTCTAATTGGATACCGAGGGGAATTTATGG

AACGTCAGTGGAGCATTTTTGACAAGAAATATTACATGTATCTAGACGTTTTTTAGGTATAGATA

CATCCATCTTTGGACAAATTTTTTTAATGTACTGAATTAACGCCGAATTGAATTATCAGCTTGCA

TGCCGGTCGATCTAGTAACATAGATGACACCGCGCGCGATAATTTATCCTAGTTTGCGCGCTATA

TTTTGTTTTCTATCGCGTATTAAATGTATAATTGCGGGACTCTAATCATAAAAACCCATCTCATA

AATAACGTCATGCATTACATGTTAATTATTACATGCTTAACGTAATTCAACAGAAATTATATGAT

AATCATCGCAAGACCGGCAACAGGATTCAATCTTAAGAAACTTTATTGCCAAATGTTTGAACGAT

CTGCTTGACTCTAGCTAGAGTCCGAACCCCAGAGTCCCGCTCAGAAGAACTCGTCAAGAAGGCGT

TAGAAGGGCGAATA

>T1294

CTGTGGACGGGGCCCCTGGGGTTCGGACTCTAGCTAGAGTCAAGCAGATCGTTCAAACATTTGGC

AATAAAGTTTCTTAAGATTGAATCCTGTTGCCGGTCTTGCGATGATTATCATATAATTTCTGTTG

AATTACGTTAAGCATGTAATAATTAACATGTAATGCATGACGTTATTTATGAGATGGGTTTTTAT

GATTAGAGTCCCGCAATTATACATTTAATACGCGATAGAAAACAAAATATAGCGCGCAAACTAGG

ATAAATTATCGCGCGCGGTGTCATCTATGTTACTAGATCGACCGGCATGCAAGCTGATAATTCAA

TTCGGCGTTAATTCAGTACATTAAAAACGTCCGCAATGTGTTATTAAGTTGTCTAAGCGTCAATT

TGTTTACACCACAATATATCCCTTTGCCTTTATGAATCGTGCTTAAATCACTAGGAGTTGCCTCC

CTCCGTACCGGAGCTCTGTCCTGTGTATACCCTGTAGCACTTGTTCCTCCTCCCCACCAACAAAT

TGTTGCTCAAAACCCTAGGCAACCCTGTTTTCCTAAAATAAAAGTTGCTGACGTTTTGTTTTCCA

CCCCAAATTGCTGCAGAAAAATACTGACCCCCAACATCATATCTTGTGTTTTTGATATCATCCTG

CTACTCTCATGATCCCCAGGACTGAAATCTGGGTCACGAAATAAACTGGGCATGACTCCAAAAGT

CATGTTTTTATCAGCAAAGCACATACATAAAAGATCAGGCAACATGTAGAAAAGCAACTCTATCA

TGAAAAGAAGCAGAGGTACTACATCGATCTGAAACATACATCATTTTTTCTTGTCAAAATGCTCC

ACTGACGTTCATAAATTCCCCTCGGTATCAATTAGAGTCTCATATTCACTCTCACTCGATCGAGC

ATGATTGACAGATGATGCACGCCAGGTAC

>T1330

GGCCGTGGCAGCAGCTGATATTCATTCGGCGTTATTCAGTACATTAAAAACGTCCGCAATGTGTT

ATTAAGTTGTCTAAGCGTCAATTT(LB264)TCGCCGACCCAGGTTTGATCCATATGGTTGACAC

GATGTATTTGTAGGAGACACATTACACATACTTAAGTGGTGGTCTGAGAGTAAAGTAGTGTGTGG

GCGTCTACGTTGTACTCTTAAAAAAACACAACTTTAATTTAAAAATGTAGACATTCAAGATTGAA

CATTCATGGAATTAGGAATTAATTAATTCTATTTCAAAATTCAAACATCTTGTAATGTAGTATAA

AGTACATGTTGTACACTTTTCAGGAACAAAAATGACTTAGTTTGGTGTTAAAGGGGTGAAAAATA

ATTTTGCAGGAATTATAGAATTATACAAATTCTGAAGATTTCGTGACGTGAGGCACCACATGGTC

TGACAAGC(1768)CGGGCCCTCTAGAGGATCCCCGGGTACCGCGAATTATCGATCATGAGCGGA

GAATTAAGGGAGTCACGTTATGACCCCCGCCGATGACGCGGGACAAGCCGTTTTACGTTTGGAAC

TGACAGAACCGCAACGTTGAAGGAGCCACTGAGCCGCGGGTTTCTGGAGTTTAATGAGCTAAGCA

CATACGTCAGAAACC

>T1427

GACTGAGGACCTGATACCAATGCGGCGGCTGCATACGCTTGATCCGGCTACCTGCCCATTCGACC

ACCAAGCGAAACATCGCATCGAGCGAGCACGTACTCGGATGGAAGCCGGTCTTGTCGATCAGGAT

GATCTGGACGAAGAGCATCAGGGGCTCGCGCCAGCCGAACTGTTCGCCAGGCTCAAGGCGCGGAT

GCCCGACGGCGAGGATCTCGTCGTGACCCACGGCGATGCCTGCTTGCCGAATATCATGGTGGAAA

ATGGCCGCTTTTCTGGATTCATCGACTGTGGCCGGCTGGGTGTGGCGGACCGCTATCAGGACATA

GCGTTGGCTACCCGTGATATTGCTGAAGAGCTTGGCGGCGAATGGGCTGACCGCTTCCTCGTGCT

TTACGGTATCGCCGCTCCCGATTCGCAGCGCATCGCCTTCTATCGCCTTCTTGACGAGTTCTTCT

GAGCGGGACTCTGGGGTTCGGACTCTAGCTAGAGTCAAGCAGATCGTTCAAACATTTGGCAATAA

AGTTTCTTAAGATTGAATCCTGTTGCCGGTCTTGCGATGATTATCATATAATTTCTGTTGAATTA

CGTTAAGCATGTAATAATTAACATGTAATGCATGACGTTATTTATGAGATGGGTTTTTATGATTA

GAGTCCCGCAATTATACATTTAATACGCGATAGAAAACAAAATATAGCGCGCAAACTAGGATAAA

TTATCGCGCGCGGTGTCATCTATGTTACTAGATCGACCGGCATGCAAGCTGATAATTCAATTCGG

CGTTAATTCAGTACATTAAAAACGTCCGCAATGTGTTATTAAGTTGTCTAAGCGTCAATTTGTTT

ACTCCGACCAGGTCGTGCAGCCGCCGGGCCCCCGAGATCCGGCCGTCGTTGCCCCAACCACCGCG

CCATCTGCAGAGGCACCAGCTCCATGGAGCTCTCCCATATGGTCGACTAGAGCCAAGCTGATCTC

CTTTGCCCCCGGAGATCACCATGGGACGACTTTCTTCTATCTCTACGATCTAGGAAGAAAGTTTC

GACGGAGAACGTGACGATACCATGTTCACCACCGATTATGAGAAGATAGCTCTTCATTCAGAAAA

GAATGCTGACCCACAGATGGGTTAAGAGAAGGCCTTACGCCGGCAAGG

>T1429

ACCCTAGCCCATTCGACCACCAAGCGAAACATCGCATCGAGCGAGCACGTACTCGGATGGAAGCC

GGTCTTGTCGATCAGGATGATCTGGACGAAGAGCATCAGGGGCTCGCGCCAGCCGAACTGTTCGC

CAGGCTCAAGGCGCGGATGCCCGACGGCGAGGATCTCGTCGTGACCCACGGCGATGCCTGCTTGC

CGAATATCATGGTGGAAAATGGCCGCTTTTCTGGATTCATCGACTGTGGCCGGCTGGGTGTGGCG

GACCGCTATCAGGACATAGCGTTGGCTACCCGTGATATTGCTGAAGAGCTTGGCGGCGAATGGGC

TGACCGCTTCCTCGTGCTTTACGGTATCGCCGCTCCCGATTCGCAGCGCATCGCCTTCTATCGCC

TTCTTGACGAGTTCTTCTGAGCGGGACTCTGGGGTTCGGACTCTAGCTAGAGTCAAGCAGATCGT

TCAAACATTTGGCAATAAAGTTTCTTAAGATTGAATCCTGTTGCCGGTCTTGCGATGATTATCAT

ATAATTTCTGTTGAATTACGTTAAGCATGTAATAATTAACATGTAATGCATGACGTTATTTATGA

GATGGGTTTTTATGATTAGAGTCCCGCAATTATACATTTAATACGCGATAGAAAACAAAATATAG

CGCGCAAACTAGGATAAATTATCGCGCGCGGTGTCATCTATGTTACTAGATCGACCGGCATGCAA

GCTGATAATTCAATTCGGCGTTAATTCAGTACATTAAAAACGTCCGCAATGTGTTATTAAGTTGT

CTAAGCGTCAATTTGTTTACACCACAATATATCCTGTCATTCAGATAGACCACGGATACTTGTTA

CCTGTAAACTATGTAAAGGAAACAGAGCAAGAAGACGTAGGACTTAAGTGGCTCTTCCCAGTCGG

CCCAAAAAGCGGAGCCCTTTTGCCAATTTCAATCAGAGGGAATAGCAGTTCCCTGCATGTTTTAG

GATGTAATCTACAAAACATTAGCAATCTTCCGAAGCATGCCTTTGCAGTTAGATTGATATCTCAG

CTCTGCATGTCTGCAGGTCGGTGGTGCTGCTGTTATATATCTTTCAATCAGAATTTCATTTTT

>T1705

GGTAGCTCAGCTGATATTCATTCGGCGTTAATTCAGTACATTAAAAACGTCCGCAATGTGTTATT

AAGTTGTCTAAGCGTCAATTTGTCTAAGCCGATGTGGTACTAAACAAGACCGTCCTGGCGCAGGC

GTGAGATGCGCATCAATCGCGGGCCTCTTTTTCTGGAAGCAAAACTTAAGGAGGAGTCTTAGTGA

AGGGCCCTCTAGAGGATCCCCGGGTACCGCGAATTATCGATCATGAGCGGAGAATTAAGGGAGTC

ACGTTATGACCCCCGCCGATGACGCGGGACAAGCCGTTTTACGTTTGGAACTGACAGAACCGCAA

CGTTGAAGGAGCCACTGAGCCGCGGGTTTCTGGAGTTTAATGAGCTAAGCACATACGTCCAGAAA

CC

>T1903

CCCTGGAACGGGGATCTGGGGTTCGGACTCTAGCTAGAGTCAAGCAGATCGTTCAAACATTTGGC

AATAAAGTTTCTTAAGATTGAATCCTGTTGCCGGTCTTGCGATGATTATCATATAATTTCTGTTG

AATTACGTTAAGCATGTAATAATTAACATGTAATGCATGACGTTATTTATGAGATGGGTTTTTAT

GATTAGAGTCCCGCAATTATACATTTAATACGCGATAGAAAACAAAATATAGCGCGCAAACTAGG

ATAAATTATCGCGCGCGGTGTCATCTATGTTACTAGATCGACCGGCATGCAAGCTGATAATTCAA

TTCGGCGTTAATACCATAAAGGAGCCTGTTACCTCCATACCGTGGAGGTTAATCCTATCGAAGGC

GCCAGTATGGGCTCTCATAATATCCCATTTTTGCCATAACTGGGGAACATTCATTCTCCTGACAT

GGATGCCCACGTACTACAATCAGGTAATTTTATTGTGTCAAGTGTTTGGCAATCATCAATTCATC

ATTGCTCGTATCAGCCAAGGAGACCATATCAAGTTATACTCACCAAATGATTTGATCCAGGTTCT

GAAGTTCAATCTTACCGAGTCCGGGCTTTTGTGTGTCTTGCCATGGCTGACAATGGCTGTATTTG

CAAATATTTCTTGTCAAAAATGCTCCACTGACGTTCCATAAATTCCCCTCGGTATCCAATTAGAG

TCTCATATTCACTCTCAACTCGATCGAGGCATGATTGAACAAGATGGATTGAC

>T1988

CCGGAACGGGATCTGGGGTTCGGACTCTAGCTAGAGTCAAGCAGATCGTTCAAACATTTGGCAAT

AAAGTTTCTTAAGATTGAATCCTGTTGCCGGTCTTGCGATGATTATCATATAATTTCTGTTGAAT

TACGTTAAGCATGTAATAATTAACATGTAATGCATGACGTTATTTATGAGATGGGTTTTTATGAT

TAGAGTCCCGCAATTATACATTTAATACGCGATAGAAAACAAAATATAGCGCGCAAACTAGGATA

AATTATCGCGCGCGGTGTCATCTATGTTACTAGATCGACCGGCATGCAAGCTGATAATTCAATTC

GGCGTTAATTCAGTACATTAAAAACGTCCGCAATGTGTTATTAAGTTGTCTAAGCGTCAATTTGT

TTACACCACAATATATCCTGGCCGTGTTGAGACTGGTGTCATCAAGCCTGGTATGATTGTTACCT

TCGGTCCTACTGGTCTGACTACTGAGGTCAAGTCTGTTGAGATGCACCATGAGGCTCTCCAGGAG

GCGCTTCCGGGTGACAATGTTGGCTTCAACGTCAAGAATGTTGCTGTGAAGGATCTCAAGCGTGG

GTTTGTGGCATCCAACTCCAAGGATGACCCTGCCAAGGAGGCTGCCAACTTCACCTCCCAGGTCA

TCATCATGAACCACCCTGGTCAGATTGGCAACGGCTACGCCCCAGTGCTGGACTGCCACACCTCC

CACATTGCAGTCAAGTTTGCTGAGCTGGTGACCAAGATCGACAGGCGATCTGGTAAGGAGCTGGA

GAAGGAGCCCAAGTTCTTGAAGAACGGTGATGCTGGTATTGTGAAGATGATTCCCACCAAGCCCA

TGGTTGTGGAGACCTTCGCTTATGAACCCTCCCCTTGGTCGTTTTGCTGTCCGTGACATGAGACA

AACAGTTTGCTGTTGGTGTCATCAAGGGCGTGAGAAGAATGGACCCAATCTGGCGCCAAGGTCAC

CAAGGGCGGTCTGC

>T1763

GGAGGCTCAGCTGATATTCATTCGGCGTTATTCAGTACATTAAAAACGTCCGCAATGTGTTATTA

AGTTGTCTAAGCGTCAATTTGTTTACACCACAATATATCCTCAGCACGGGACGCTTGTCGACCAG

GTGCCACAGCCAGTCAGGGTACTCTGCATCTGGCTGGATCTTTGGATCAGAACCCTCCTTCAGGA

TATTTGCACCAAACACAGTTGTGCTCTTCATTTCCTTGCTGAGCACAGGTTTAGCATCAGCTGTG

CCACCTTTTCCACCCTTCTTTCCCTTGGCTGCAACTGCAAGGCCTCTGGCCCCGATCAACTGAGC

TGCATCTCTTGGTATAATTCCTTGCTTCAATACTCTTGTCCAAGGCATTGCCATTCCTGTGTTGA

TGATTTTTTAACTGCACAAATCAATCACCAGAACGAACTGTCAACATATAAAACAGAAAACAACA

ACAATTCTGAACAAAGTTTAGAATCTGAAAAACAAGTTAGTCATTTAACTGGAAAGACAACGTGA

GTACATAGATGTCAAAGGAGTATGTTGACAAGAGCTGCATATAACATAGGCCGGTTAATTCTTCC

CCAGAAGCTAGCATGCGTGTGTCAGCCGTGATGTATGTGTGCTAGCAGGTTACAGCTTGCATGCT

GGCCTAAGGAGCTAGCCAGGCCACCATGTTGGTCGGGCCCTCTAGAGGATCCCCGGGTACCGCGA

ATTATCGATCATGAGCGGAGAATTAAGGGAGTCACGTTATGACCCCCGCCGATGACGCGGGACAA

GCCGTTTTACGTTTGGAACTGACAGAACCGCAACGTTGAAGGAGCCACTGAGCCGCGGGTTTCTG

GAGTTTAATGAGCTAAGCACATACGTCCAGAAAACC

>T2603

ATCAAACCAACTAGACACACACATTGAGTCAGCAGGAGACTTGAGAGGAGGAGTGGTGTAAACAA

ATTGACGCTTAGACAACTTAATAACACATTGCGGACGTTTTTAATGTACTGAATTAACGCAGAAT

TGAATTATCAGCTTGCATTCCGGTCGATCTAGTAACATAGATGACACCGCGCGCGATAATTTATC

CTAGTTTGCGCGCTATATTTTGTTTTTTATCGGTTTATTAAATGTATAAGTGGGGGTCCATAATC

CTACAAACCCATCAAAAAAATAACGTCAGGCTCTACATGTTAAATGTAGCGGGGGTCAGGCAATT

CAACAGAAATTATATGTGAATCAAGCAAGACTCAGTACAGGAATTCCTGCTCAGAGGCTTTATGG

CCAAAGGTTTGAACGATTTGCTAGACTCGAGCGAGAGTCTCGAACTCTAGATTCCCAACCGCG

>T2638

TTTTTATGATTAGAGTCCCGCAATTATACATTTAATACGCGATAGAAAACAAAATATAGCGCGCA

AACTAGGATAAATTATCGCGCGCGGTGTCATCTATGTTACTAGATCGACCGGCATGCAAGCTGAT

AATTCAATTCGGCGTTAATTCAGTACATTAAAAACGTCCGCAATGTGTTATTAAGTTGTCTAAGC

GTCAATTTGTTTACACCACAATATATCCTGTGAGGTGCCGAACGGTTGACTTTTTGACCAGGGGA

GCGCCATCAACCGAGGACACGCCACACAGCTCGCCTGCCTAGTAAGATACAACTACCATAGCCGC

ATGATAATTCGGCTTCTCAGCGATCCACGTCAGCGTTTTGTACTGACGCGGATGTTTTTCTGGTT

TTGCCAGGGTGGCGGGTGCTGTGTTAGTTTTCTCGCTGBDACGGTGGGGCCCTCTAGAGGATCCC

CGGGTACCGCGAATTATCGATCATGAGCGGAGAATTAAGGGAGTCACGTTATGACCCCCGCCGAT

GACGCGGGACAAGCCGTTTTACGTTTGGAACTGACAGAACCGCAACGTTGAAGGAGCCAC

>T2662

CATGCAAAGAGAAGTGTAGCTATACTCCAGTGTTGCTTCGATTTGCAAGCGCCCTGGGATCCCGG

TGGTTTGTCACAATGTCGGCTACAAGGAATGCCTCAATCCAGAACTGGTTCTAATATGGTTTCGA

CTCACCCCGCGCACATCATGGGACCCTGGTGGTTTGACATGGAGTCGGCTTGAGGGCAAGCCGCA

TTTTATGGAGGGGGGCTTGTCAGCGTCTCTGTCTACACGGACCCATCGACCAGCTTCCTTAGGCA

CGGCTCCACCTGTAAGGTAAACAAATTGACGCTTAGACAACTTAATAACACATTGCGGACGTTTT

TAATGTACTGAATTAACGCCGAATTGAATTATCAGCTTGCATGCCGGTCGATCTAGTAACATAGA

TGACACCGCGCGCGATAATTTATCCTAGTTTGCGCGCTATATTTTGTTTTCTATCGCGTATTAAA

TGTATAATTGCGGGACTCTAATCATAAAAACCCATCTCATAAATAACGTCATGCATTACATGTTA

ATTATTACATGCTTAACGTAATT

>T2725

AGTAATAAAATTATCACATACTTGTCTCATTTTTATAGATTCATGGTGCAAAGTAAATAGGACTA

GAATCAATCACTGCCATTCCAAGTGGCCGTGTGTGTGCTGCCAAGTTAGTTGCAAACCAAATGGA

TGCCTATATTGCGAAGAAAAGAAAGGCAGGTACATAGCTATAATCCAAACAACAACAACAACTTG

CCAAAAAGTAAGCTAATTATATCCCTTGTCATGCCAAAAGAATTAGTTAGGCTACTTGAGCATCA

AACCAACTAGACACACACATCGAGACAGCAGGAGACTTGAGAGGAGGAGTGGTGTAAACAAATTG

ACGCTTAGACAACTTAATAACACATTGCGGACGTTTTTAATGTACTGAATTAACGCCGAATTGAA

TTATCAGCTTGCATGCCGGTCGATCTAGTAACATAGATGACACCGCGCGCGATAATTTATCCTAG

TTTGCGCGCTATATTTTGTTTTCTATCGCGTATTAAATGTATAATTGCGGGACTCTAATCATAAA

AACCCATCTCATAAATAACG

>T2738

CATACTTGTCTCATTTTTATAGATTCATGGTGCAAAGTAAATAGGACTAGAATCAATCACTGCCA

TTCCAAGTGGCCGTGTGTGTGCTGCCAAGTTAGTTGCAAACCAAATGGATGCCTATATTGCGAAG

AAAAGAAAGGCAGGTACATAGCTATAATCCAAACAACAACAACAACTTGCCAAAAAGTAAGCTAA

TTATATCCCTTGTCATGCCAAAAGAATTAGTTAGGCTACTTGAGCATCAAACCAACTAGACACAC

ACATCGAGACAGCAGGAGACTTGAGAGGAGGAGTGGTGTAAACAAATTGACGCTTAGACAACTTA

ATAACACATTGCGGACGTTTTTAATGTACTGAATTAACGCCGAATTGAATTATCAGCTTGCATGC

CGGTCGATCTAGTAACATAGATGACACCGCGCGCGATAATTTATCCTAGTTTGCGCGCTATATTT

TGTTTTCTATCGCGTATTAAATGTATAATTGCGGGACTCTAATCATAAAAACCCATCTCATAAAT

AACGTCATGCATTACATGTT

>T2740

TTTGCTGCCTCGTCTTGGAGTTCATTCAGGGCACCGGACAGGTCGGTCTTGACAAAAAGAACCGG

GCGCCCCTGCGCTGACAGCCGGAACACGGCGGCATCAGAGCAGCCGATTGTCTGTTGTGCCCAGT

CATAGCCGAATAGCCTCTCCACCCAAGCGGCCGGAGAACCTGCGTGCAATCCATCTTGTTCAATC

ATGCCTCGATCGAGTTGAGAGTGAATATGAGACTCTAATTGGATACCGAGGGGAATTTATGGAAC

GTCAGTGGAGCATTTTTGACAAGAAATATTTGCTAGCTGATAGTGACCTTAGGCGACTTTTGAAC

GCGCAATAATGGTTTCTGACGTATGTGCTTAGCTCATTAAACTCCAGAAACCCGCGGCTCAGTGG

CTCCTTCAACGTTGCGGTTCTGTCAGTTCCAAACGTAAAACGGCTTGTCCCGCGTCATCGGCGGT

GCTGCCTCGTCTTGGAGTTCATTCAGGGCACCGGACAGGTCGGTCTTGACAAAAAGAACCGGGCG

CCCATGCGCCGACAGCCGGAACACGGCGGCATCAGAGCAGCCGATTGTGAGTTGTGCCCAGTAAC

AGAAATTATATGATAATCATCGCAAGACCGGCAACAGGATTCAATCTTAAGAAACTTTATGCCAA

ATGTTGAACGATCGA

>T2939

CATCCATCTTGTTCAATCATGCCTCGATCGAGTTGAGAGTGAATATGAGACTCTAATTGGATACC

GAGGGGAATTTATGGAACGTCAGTGGAGCATTTTTGACAAGAAATATTTGCTAGCTGATAGTGAC

CTTAGGCGACTTTTGAACGCGCAATAATGGTTTCTGACGTATGTGCTTAGCTCATTAAACTCCAG

AAACCCGCGGCTCAGTGGCTCCTTCAACGTTGCGGTTCTGTCAGTTCCAAACGTAAAACGGCTTG

TCCCGCGTCATCGGCGGGGGTCATAACGTGACTCCCTTAATTCTCCGCTCATGATCGATAATTCG

CGGTACCCGGGGATCCTCTAGAGGGCCCGACAAGATAAGCGAGTGGCCATCAATTTTAGCCAACA

TCCTTATAGGGAAAACCTCGACAATGTTCTTTTTATCCTTAGACAACTTAATAACACATTGCGGA

CGTTTTTAATGTACTGAATTAACGCCGAATTGAATTATCAGCTTGCATGCCGGTCGATCTAGTAA

CATAGATGACACCGCGCGCGATAATTTATCCTAGTTTGCGCGCTATATTTTGTTTTCTATCGCGT

ATTAAATGTATAATTGCGGGACTCTAATCATAAAAACCCATCTCATAAATAACGTCATGCATTAC

ATGTTAATTATTACATGCTTAACGTAATTCAACAGAAATTATATGATAATCATCGCAAGACCGGC

AACAGGATTCAATCTTAAGAAACTTTATTGCCAAATGTTTGAACGATCTGCTTGACTCTAGCTAG

AGTCCGAACCCCAGATCCGTTCAGG

>T3162

GGATAACAATTTCACACAGGAAACAGCTATGACCATGATTACGCCAAGCTCTAGCTAGAGGTTGA

CGGTATACAGACATGATAAGATACATTGATGAGTTTGGACAAACCACAACTAGAATGCAGTGAAA

AAAATGCTTTATTTGTGAAATTTGTGATGCTATTGCTTTATTTGTAACCATTATAAGCTGCAATA

AACAAGTTGGGGTGGGCGAAGAACTCCAGCATGAGATCCCCGCGCTGGAGGATCATCCAGCCGGC

GTCCCGGAAAACGATTCCGAAGCCCAACCTTTCATAGAAGGCGGCGGTGGAATCGAAATCTCGTG

ATGGCAGGTTGGGCGTCGCTTGGTCGGTCATTTCGAACCCCAGAGTCCCGCTCAGAAGAACTCGT

CAAGAAGGCGATAGAAGGCGATGCGCTGCGAATCGGGAGCGGCGATACCGTAAAGCACGAGGAAG

CGGTCAGCTCATCGCCGCCACTTCA
